# Sexuality after breast cancer: cultural specificities of Tunisian population

**DOI:** 10.11604/pamj.2016.25.17.10399

**Published:** 2016-09-20

**Authors:** Mnif Leila, Charfi Nada, Chaabene Kais, Masmoudi Jawaher

**Affiliations:** 1 Psychiatry Department, Hedi Chaker Hospital, Sfax, Tunisia; 2 Gynecology Department, Hedi Chaker Hospital, Faculty of Medicine of Sfax, Tunisia

**Keywords:** Breast cancer, sexual functioning, satisfaction, quality life, body image, culture, depression, anxiety

## Abstract

**Introduction:**

Women’s sexuality may be particularly affected after breast cancer. The objectives of this study were to evaluate the changes in sexual life after treatment of breast cancer in Tunisian women and to identify the influence of demographic and clinical factors on sexuality.

**Methods:**

We recruited 50 patients who were in remission for at least 3 months after initial treatment of breast cancer. Sexuality and body image were evaluated using the Arabic version of the specific scale of breast cancer QLQ-BR23. Screening for emotional disorders has been done with the Arabic version of HAD scale (Hospital anxiety and depression scale).

**Results:**

Patients had poor sexual functioning and sexual satisfaction and the mean scores were respectively 45.3% and 43.9%. Only menopausal status and sexual difficulties in the partner was significantly related to poorer sexual satisfaction (p respectively 0.018 and 0.014). According to the HAD scale, 42% of patients had anxiety and 44% had depression. The sexual satisfaction was statistically associated with the presence of anxiety symptoms (p=0.0003).

**Conclusion:**

Results suggest that the psychological side and the sexual difficulties in partner are the most important factors that appear to be involved in sexual satisfaction of Tunisian women after breast cancer. So, those factors need to be taken into account in therapeutic process and psychological counseling to maintain and enhance patient’s psychological well-being.

## Introduction

It is widely recognized that women’s sexuality can be particularly complex after breast cancer, with sexual changes often becoming the most problematic aspect of a woman’s life. The impact of such changes can last for many years after successful treatment. Sexuality after breast cancer is considered like an important aspect of quality of life [1]. Factors related to treatment and its clinical implications may contribute to these problems. Women's intrapsychic experience of changes to sexuality includes a fear of loss of fertility, negative body image, feelings of sexual unattractiveness, loss of femininity, depression and anxiety, as well as alterations to a sense of sexual self [2]. A growing number of studies have been conducted that explore the sexual experiences in women presented breast cancer from diverse ethnocultural groups [3]. The meaning of breast cancer for women’s differs from a culture to another. The study aims to evaluate the changes in sexual life after treatment of breast cancer in Tunisian women and to identify the influence of demographic and clinical factors on sexuality.

## Methods

Breast cancer patient’s post-primary treatment participated in the present study. Criteria for participating included: being in remission for at least 3 months, not receiving any oncologic therapy at the moment of the study and being able to communicate. Women with metastatic cancer were excluded. Those patients were previously referred to the Department of Gynecology in Sfax University Hospital for breast surgery during the period from 2010 to 2013, and then they had all received adjuvant therapy. They were identified at the hospital patient registration department. The phone number of each patient was obtained to contact her by telephone. The selected patients were invited to attend the hospital to collaborate to the study. The study's purpose was explained to each patient and consent was obtained orally. Only 50 eligible patients agreed to participate. Among the other contacted women, 15 were not found, 5 had passed away and 10 did not consent to the study. A sociodemographic form, composed by questions related to personal characteristics (age, socioeconomic level, hormonal status, sexual problems after cancer…) and their partners characteristics (sexual problems, marital relationship) was utilized for data collection. Clinical data (cancer staging, surgical therapy, adjuvant therapy) were collected from patients’ files. All the files and medical records were consulted after patient’s permission. Sexuality and body image was evaluated using the specific scale of breast cancer QLQ-BR23 [4], which is an additional module to the European Organization for Research and Treatment of Cancer (EORTC) QLQ-C30 questionnaire [5]. An Arabic translation of this questionnaire is already approved by the authors of the instrument [6]. Screening for emotional disorders has been done using the HAD scale (Hospital anxiety and depression scale), which is a structured self-administered questionnaire of 14 items developed by Zigmond and Snaith in 1983 [7]. We also used a dialectal Arabic version of the questionnaire which is validated [8]. The data were analysed using the SPSS in its 11th version. Descriptive statistics were used to analyze demographic and clinical data. The mean QLQ-BR23 sexual functioning and satisfaction cores and sociodemographic and clinical variables were compared using ANOVA (General Linear Model). The correlations between sexual functioning and satisfaction and body image were calculated using Spearman’s Correlation Coefficient and a multiple regression model. The domain of body image (QLQ-C30) variable was the predictor variable. The t-student test was used to compare sexuality and body image according to emotional disorders. P < 0.05 was considered statistically significant.

## Results

### Demographic characteristics of the study sample

The average age of patients studied was 52.06 years with extremes of 32 and 77 years (SD = 10.07 years). The grouping by age showed that 68% of patients were aged 40-60 years. The socioeconomic level was low in 44% of cases. The majority of our patients were married (94%).

### Menopause

The majority of patients were postmenopausal women (90%) during the evaluation. Of the 25 not yet menopausal patients at diagnosis, 16 (64%) had presented a menopause induced by the effect of adjuvant therapy.

### Clinical and therapeutic characteristics

**Age of patient at diagnosis:** the mean age at time of primary diagnosis was 49.66 years with extremes of 30 and 70 years (SD= 10.2 years). Most patients (86%) were over the age of 40 years at the time of primary diagnosis of breast cancer.

**Duration of remission:** the average duration of patients’ remission was 17.4 months with a standard deviation of .17 months with a minimum of 3 months and a maximum of 40 months (3 years and 4 months).

**Breast surgery and adjuvant therapy:** forty-one patients (82%) were treated with mastectomy type Patey. Breast conserving therapy with lumpectomy was performed in 9 patients (18%). All the patients received adjuvant chemoradiotherapy.

**Endocrine therapy:** endocrine therapy, represented by anti-estrogens, was shown in 32 patients (64%).

**Hormonal status after cancer treatment:** among the 25 patients not yet menopausal at diagnosis, 16 (64%) had presented menopause induced by the effect of adjuvant therapy.

### Sexuality after breast cancer

**Sexual problems after breast cancer:** a decrease in the frequency of sexual intercourses after cancer was reported by 53.2% of patients (N = 25) sexually active. The diminished sense of sexual attractiveness was observed in 80.9% of patients and 42.5% of women had presented dyspareunia (Table 1).

**Table 1:** Frequency of sexual problems in the two samples

Variables	N	%
Decreased frequency of intercourses	25/47	53.2
Diminished sense of sexual attractiveness	38/47	80.9
Dyspareunia	20/47	42.5
Vaginal dryness	19/47	40.4

**Partner sexuality:** women interviewed reported that eighteen partners (38.3%) have changed in their sexual function after the illness: decreased libido in 55.6% of cases (N = 10), sexual dissatisfaction in 27.8% of cases (N = 5) and finally erectile dysfunction in 16.7% of cases (N = 3).

**Changes in the couple's relationship after cancer:** among the subgroup of married women (N = 47), the marital relationship before illness was good for 43 patients (91.48%) and confrontational for 4 patients (8.52%). Thirty-two patients (68.08%) among 47 married describe a change in their marital relationship after cancer (Table 2).

**Table 2:** Marital relationship changes after cancer

Marital relationship changes	N	%
Separation	2	6.2
Deterioration	5	15.6
Improvement after difficulties at the beginning	7	21.9
Strengthening	18	**56.3**
Total	32	100

**Sexuality at the QLQ-BR23:** the patients had poor sexual functioning and sexual satisfaction and the mean scores were respectively 45.3% and 43.9% (Table 3).

**Table 3:** Mean scores of functional scales at QLQ-BR23

*QLQ-BR23 variable*	*N*	*Patients Mean+/- SD*
Functional scales		
Body image	50	47.6±26.4
Sexual functioning	47	45.3±22.1
Sexual satisfaction	47	43.9±25.1

N: number, SD: standard deviation

**Predictors of sexual problems:** additional analyses indicated that menopausal status and sexual difficulties in the partner was significantly related to poorer sexual satisfaction (p respectively 0.018 and 0.014) (Table 4).

**Table 4:** Predictive factors of sexual problems

*Variables*		*Sexual functioning*		*Sexual satisfaction*	
		*M*	*p*	*M*	*P*
Age (years)	<50	49±22	0.415	51±27	0.192
	50-59	40±20		39±21	
	≥60	44±24		36±2H	
Hormonal status	Premenopausal	54±15	0.329	74±16	**0.018**
	Postmenopausal	44±22		41±23	
Endocrine therapy	Yes	47±21	0.278	45±23	0.5
	No	39±23		39±28	
Breast surgery	Lumpectomy	57±22	0.095	51±24	0.302
	Mastectomy	42±21		42±25	
Vaginal dryness	Yes	46±19	0.774	42±18	0.68
	No	44±24		45±28	
Dyspareunia	Yes	50±20	0.201	45±20	0.695
	No	42±22		43±27	
Partner sexual problems	Yes	38±28	0.114	31±29	**0.014**
	No	49±16		51±19	

M: mean score

**Impact of body image on sexuality:** the mean score of body image was 47.7% and its alteration had no significant impact on the themes of sexuality (Table 5).

**Table 5:** Correlations between body image and sexuality

Variables	Body image	
	*r*	P
Sexual functioning	**0.096**	0,523
Sexual satisfaction	**0.145**	0,331

### Sexuality and emotional disorders

**Anxiety and depression scale: HAD:** according to a categorical approach referring to the HAD scale, 42% of patients in our study (N = 21) had anxiety and 44% (N = 22) had depression.

**The impact of emotional state on sexuality:** there was a statistically significant difference between anxious patients and those with normal mood for item sexual satisfaction. The perception of body image was more negative for anxiety and depression patients, compared with patients with normal mood (Table 6).

**Table 6:** Correlations between emotional disorders and sexuality

*QLQ BR-23*	*Anxiety*				*Depression*		
	A+	A-	p	D+	D-	p
	M±SD	M±SD		M±SD	M±SD	
Functional scales						
Sexual functioning	41±22.47	48±21.91	0.111	39±21.13	49±22.16	0.096
Sexual satisfaction	36±24.57	48±24.81	**0.0003**	36±23.93	49±25.09	0.085
Body image	34±25.7	57±22.75	**0.002**	33±20.4	58±25.45	**0.0002**

M: mean, SD: standard deviation, A+: anxiety, A-: absence of anxiety, D+: depression, D-: absence of depression

## Discussion

Thanks to early detection and treatment improvement, the mortality rate from Breast Cancer has declined over the past 20 years [9, 10]. Approximately 91% of women with breast cancer now survive for more than 5years [10]. Despite improved survival rates, the literature describes a wide range of difficulties in patients’ day-to-day life arising from breast cancer and associated treatment [2, 11]. Survivors face a multitude of challenges, including issues related to fertility and sexuality [12]. Changing the frequency of sex was a common disorder reported by our patients. Indeed, 53.2% of sexually active women had reported a decrease in the frequency of reporting sexual intercourse. The number of sexual intercourse had increased for a minority of women (8.5%). This variability reflects the impact of breast cancer on sexual activity which differs from one couple to another. For some, sexuality remains important but the recovery of sexual activity is difficult anyway. For others, cancer allows some women and even some couples, to "use" the cancerous disease and its treatments to end a heavy sexuality, experienced as a constraint [13]. Fahami F reported that 47% of Iranian women who presented in the majority of cases, a breast cancer, had sexual dysfunction [14]. There was a significant correlation between sexual functioning and age, occupation, educational level, and treatment duration. There was also a significant correlation between the stage of disease, primary disorder (0.003), and recent disorder (0.028). According to various studies, oriental women reported more sexual dysfunctions compared to women in Western countries [15, 16].

Sexual dysfunctions are common to gynecologic cancer treatment, where most women experienced impaired sexual function [17]. Zeng YC study revealed that sexual dysfunction was an important concern among Chinese women with gynecologic cancer (62.2%) [18]. The rate of sexual inactivity (70.5%) was relatively high. Reasons for sexual inactivity were related to worry about possibly weakening the potency of treatment (46.5%), fear of cancer recurrence (41.1%), and lack of sexual interest (31%) [18]. In assessing the quality of sexual life, we have found poor average scores on functional scales QLQ-BR23 regardless of age. Speer and al [19] had found that breast cancer sexual functioning was significantly poorer than normal controls in all areas but desire. Breast cancer confronts the woman to complex personal and marital disorders related to the sexual sphere. It is likely to affect three main areas of sexuality: sexual identity, sexual function and sexual relationship [20]. The diminished sense of sexual attractiveness was also found in the majority of our patients (76%). Negative perceptions of body image among breast cancer survivors include dissatisfaction with appearance, perceived loss of femininity and body integrity, reluctance to look at one’s self naked, feeling less sexually attractive, self-consciousness about appearance, and dissatisfaction with surgical scars [21]. This result can be explained by the sensitivity of the affected organ and the physical and psychological impact of illness and treatment received on body image. But, all married women were sexually active, in this study. This may be, reflect the concept of “sexual obedience” in our culture which is based on the social duty of women to meet the husband’s sexual needs.

Our study showed a statistically significant correlation between alteration of sexual satisfaction and perception of body image with the presence of anxiety and depression. In the literature, breast cancer diagnosis and treatment is reported to lead patients to psychological problems such as anxiety, depression, anger, uncertainty about the future, hopelessness, helplessness, fear of relapse, decreased self-esteem, body image distortion, fear of losing the feminine characteristics and fear of death [22]. The meta-synthesis performed by Berterö [23], revealed 4 aspects of the self-affected by the diagnosis of breast cancer and its treatment: awareness of their own mortality, living with an uncertain certainty, attachment validation, and redefinition of self. In addition, anxious and / or depressed women had more altered body image. This maintains the relationship between the negative body image, deterioration of sexual life, anxiety and depression [21, 24]. However, in our study the functioning and sexual satisfaction of patients were independent of altered body image. Sexual self-schema appears to be an important concept in predicting sexual dysfunction in others study [25], but, the relationship of good body image with the resumption of sexual relations and sexual dysfunction is inconsistent. While the majority of studies have found this relationship, a few have not [21]. Indeed, it is suggested that the body image and sexuality are more affected by breast cancer during the first year of survival [21, 26]. The good marital relationship could explain our results since a negative evolution of the marital relationship after cancer had affected only 21.8% of cases. Bertero study's [23] highlights the existential process that women of many cultures move through as they incorporate the meaning of breast cancer into their lives. Spirituality was reported to be the main coping mechanism used during all phases of the cancer experience [27]. In fact, spirituality may improve acceptance of body alterations. Body image was the less altered dimension, in QLQ-BR23 functional scales in our study since believer’s subjects represent the majority of our society.

The emergence of sexual dysfunction type dyspareunia (61.5%) and vaginal dryness (61.3%) could justify the decrease in the frequency of intercourses in the group of our patients. Speer and al. [19] had observed that women who were older had significantly more concerns about vaginal lubrication and pain. This type of problem is particularly common after adjuvant chemotherapy. It is considered in the literature as the most responsible for the problems of lubrication and dyspareunia [28]. A review of the literature suggests that breast cancer patients who undergo chemotherapy are at high risk for sexual dysfunction after treatment [29]. Chemotherapy has been shown to be associated with short and long-term effects on sexual functioning and quality of life in breast cancer, and it is anticipated that this would extend to gynecologic cancers also [25]. Common effects of chemotherapy on ovarian function include temporary or permanent amenorrhea due to premature ovarian failure. Rates of amenorrhea can reach 80% in women under 40 years of age with poor prognosis tumors who are prescribed more aggressive regimens [30]. The addition of endocrine treatments to chemotherapy in breast cancer does not appear to affect levels of sexual functioning, although this may depend on the age of the woman [25]. In our patients, there was no relation between type of surgery and sexual functioning or satisfaction. But Sun and al. [31] had reported that patients in the reconstruction after total mastectomy group had significantly better outcomes on the sexual scale of the European Organization for Research and Treatment of Cancer Quality of Life Questionnaire breast cancer-specific module and arm symptoms than the total mastectomy group.

In contrast, Thors [29] had found in his study that there is little evidence of a link between type of surgical treatment (eg, lumpectomy versus mastectomy) or treatment with tamoxifen and sexual functioning outcomes. Binney observed in North Carolina that there was no statistically significant relationship between the sexual functioning of women with breast cancer and the type of treatment (chemotherapy, radiotherapy, and surgery) [32]. The study results of Sbitti and al [33] on 125 women with breast cancer in Morocco indicated that there was no statistical relationship between sexual dysfunction and age, level of education, disease stage, and type of treatment. Fahmi study results showed no significant relationship between cancer type and type of sexual disorder: primary (0.403) or recent (0.416) [14]. In our study, all the patients had received chemotherapy, and sexuality was independent of surgical or endocrine treatment. Additional analyses indicated that menopausal status and sexual difficulties in the partner were significantly related to poorer sexual satisfaction (p respectively 0.018 and 0.014). These two factors were probably for women the warning signs of the end of their sexuality, in a society where divorce is poorly accepted and menopause is designed by “despair age”. Several studies [34, 35] reported the installation of sexual difficulties in the majority of partners of women with cancer. Both patient and partner are dealing with concerns about survival [36]. The breast cancer level of relationship distress was the most significant variable affecting arousal, orgasm, lubrication, satisfaction, and sexual pain [19]. Depression and having traditional role preferences are the most important determinants of lower sexual desire [19]. Finally, the quality of a woman's partnered relationship consistently predicts sexual health post-breast cancer -specific- reinforcing the importance of recognizing the intersubjective nature of issues surrounding breast cancer and sexuality [37]. It is therefore essential to involve the spouse in the care of the patient and encourage couples to improve communication. Information provision can also prevent or ameliorate distress [38]. Communication with women about sexual issues is vital, but evidence suggests that is lacking [25]. Ussher JM reported that substantial proportion of patients (68%) search for information on physical changes, sexual response, relationship issues, psychological consequences, and body image or identity [38]. These findings provide clinicians and cancer organizations with specific suggestions about sexual information needs after breast cancer and the modalities which are preferred, to prevent and ameliorate distress [38]. Martin described how exercise facilitated counseling in survivors of breast and prostate cancer, by creating mutual aid and trust, and counseling helped participants with self-identity, sexuality, and returning to normalcy [39].

## Conclusion

Considering the specific impact on sexuality and partner relationship of non-metastatic breast cancer and its treatments, it seems fundamental to educate physicians and caregivers to this dimension, and facilitate dialogue on this topic. The physical determiner does not seem to resound negatively on women's sexuality while the psychological side most likely intervenes in sexual satisfaction. Hence the importance of psycho - oncological care that will accompany the patient and her spouse to help them anticipate the changes related to the disease and its treatments.

### What is known about this topic

- Breast cancer affects women’s feelings of sexuality;
- After breast cancer, changes to sexual well-being are associated with physical adverse effects;
- Sexuality relates directly to women’s well-being after breast cancer.

### What this study adds

- After breast cancer, health care professionals can play an important role in discussing sexuality with patient’s and her partner;
- The presence of anxiety and depression in patients with breast cancer lead to the alteration of sexual satisfaction;
- We suggest that a consultation with a psychologist, focusing on issues such as body image, changes in sexuality and communication with the partner, is required as standard of care in the counselling of women after primary treatment of breast cancer.
